# The association of artificial sweeteners intake and risk of cancer: an umbrella meta-analysis

**DOI:** 10.3389/fmed.2025.1647178

**Published:** 2025-09-08

**Authors:** Ahmed Abu-Zaid, Emad Kutbi, Nawal Alshammari, Abdullah Nasser AlJurayyan, Heba M. Adly, Saleh A. K. Saleh, Saeed Baradwan, Madiha Jamal, Feham Peer-Zada, Shaimaa Mohamed, Huda Syed, Rania Salah Ahmed, Mohammed Abuzaid, Osama Alomar

**Affiliations:** ^1^College of Medicine, Alfaisal University, Riyadh, Saudi Arabia; ^2^Department of Biorepository, Research Center, King Fahad Medical City, Riyadh, Saudi Arabia; ^3^Livestock and Fisheries Development Program, Biotechnology Sector, National Fisheries Development Program, Riyadh, Saudi Arabia; ^4^Department of Community Medicine and Pilgrims Healthcare, College of Medicine, Umm Al-Qura University, Makkah, Saudi Arabia; ^5^Directorate of Institutional Excellence, Batterjee Medical College, Jeddah, Saudi Arabia; ^6^Department of Obstetrics and Gynecology, King Faisal Specialist Hospital and Research Center, Jeddah, Saudi Arabia; ^7^Department of Obstetrics and Gynecology, Al Birk General Hospital, Al Birk, Saudi Arabia; ^8^Department of Obstetrics and Gynecology, King Faisal Specialist Hospital and Research Center, Riyadh, Saudi Arabia

**Keywords:** cancer, umbrella meta-analysis, artificial sweeteners, low-calorie sweeteners, risk

## Abstract

**Background:**

Previous meta-analyses exploring the relationship between artificial sweetener consumption and cancer risk have shown inconsistent results. To address these discrepancies, we conducted an umbrella review of systematic reviews and meta-analyses of observational studies.

**Methods:**

We systematically searched PubMed, Scopus, and Web of Science up to January 2025. Pooled relative risks (RRs) and 95% confidence intervals (CIs) were recalculated using a random-effects model. Subgroup and sensitivity analyses assessed the robustness of findings.

**Results:**

Ten meta-analyses comprising 35 datasets were included. Based on the AMSTAR 2 tool, three reviews were rated as high quality, two as moderate, and five as low. Overall, artificial sweetener intake was not significantly associated with cancer risk (RR: 0.99; 95% CI: 0.96–1.01). This finding reflects the effect of various sweeteners grouped together and should not be extrapolated to individual compounds. Sensitivity analyses confirmed the robustness of findings, with no publication bias detected. Across study designs—prospective (RR: 1.00; 95% CI: 0.92–1.08), case-control (RR: 0.94; 95% CI: 0.86–1.03), and cohort–case-control (RR: 0.96; 95% CI: 0.77–1.14)—associations were consistently non-significant. By sweetener source, no significant associations emerged for artificially sweetened beverages (RR: 0.98; 95% CI: 0.96–1.01) or artificial sweeteners overall (RR: 1.00; 95% CI: 0.94–1.06), both with low heterogeneity. Results were consistent across RR, odds ratio, and hazard ratio. By cancer type, no significant associations were found except for gynecological cancers, where higher intake was linked to reduced risk (RR: 0.87; 95% CI: 0.79–0.96; *I*^2^ = 0%).

**Conclusion:**

The findings of this umbrella review do not support a significant association between artificial sweetener intake and overall cancer risk, with possible protective effects limited to gynecological cancers. Findings were consistent across study types and robust to sensitivity analyses.

## Introduction

Cancer has emerged as a significant global health challenge, with an estimated 23.6 million new cases and 10.0 million deaths from cancer worldwide in 2019, representing a 26.3% increase in new cases and a 20.9% increase in deaths compared to previous years (1, 2). The burden of cancer is predicted to continue to rise for at least the next two decades (2). Studies have shown that a high-sugar diet can contribute to the development of obesity and cardiovascular disease, either directly or indirectly (3). Similarly, research has linked a high-sugar diet to increased rates of cancer (4). Consequently, sweeteners have become a more popular alternative to sugar in food and beverages in recent decades (5).

The utilization of artificial sweeteners as a low-calorie replacement for sugar is prevalent in various food and beverage products (6). Artificial sweeteners are utilized in minute quantities to provide sweetness without adding calories, as they are significantly sweeter than sugar. Nonetheless, there is a persistent debate surrounding the safety and potential health impacts of artificial sweeteners (7). Artificially sweetened beverages (ASBs) refer to non-alcoholic drinks that contain low-calorie sweeteners (LCSs) as a substitute for sugar, offering a sweet taste without added calories (8, 9). Common LCSs used in ASBs are aspartame, acesulfame-K, saccharin, sucralose, and neotame. ASBs are often marketed as a healthier option to sugar-sweetened beverages (SSBs) and have become popular due to growing concerns regarding the detrimental health effects of excessive sugar consumption (10). However, artificial sweeteners are also commonly found in a wide range of other processed foods, including yogurts, desserts, chewing gums, baked goods, and even pharmaceuticals.

It is important to clarify the terminology used when discussing these products. The term “low-calorie sweeteners” (LCSs) or “non-nutritive sweeteners” (NNSs) represents a broad category of sugar substitutes. This category includes highly intense “artificial sweeteners”, which are synthetically produced compounds such as aspartame, acesulfame-K, saccharin, and sucralose. It also includes sweeteners derived from natural sources, like stevia, and sugar alcohols. Although these compounds differ in their origin and biological pathways, they are often grouped together in nutritional research and food manufacturing due to their shared function of providing sweetness with minimal to no caloric value. For the purpose of this umbrella review, we use the term “artificial sweeteners” inclusively to encompass the broad range of compounds examined in the source meta-analyses, reflecting the comprehensive scope of our search strategy. This approach is necessary because the included studies often do not disaggregate their findings by specific sweetener type (11).

Research examining the safety and efficacy of artificial sweeteners has produced conflicting results. While some studies have reported that these sweeteners are safe and beneficial for reducing sugar intake and assisting with weight management (12), other studies have expressed concerns regarding the possible negative impacts on health, including a potential rise in the risk of cancer (8). In a recent meta-analysis, the consumption of artificially sweetened soda, which is considered an ASB, was found to increase the risk of liver cancer by 28% (13). There has been a growing concern regarding the role of sweetened beverages (SBs) in increasing the risk of pancreatic cancer (PC) (14). A recent study investigated the association between artificial sweetener use, including aspartame, and cancer risk, and results showed that high consumption of other artificial sweeteners was linked to colorectal and stomach cancer among participants with diabetes (15). Observational epidemiological studies conducted previously have yielded conflicting results regarding whether the consumption of ASB increases the risk of gastrointestinal (GI) cancer (16).

The available evidence indicates that there is a logical biological connection between the consumption of sweet beverages and the development of cancer. Nevertheless, it is important to note that various types of artificial sweeteners may operate through distinct mechanisms and have varying degrees of involvement in the onset of cancer (17). However, the potential long-term health risks associated with ASB intake, particularly their possible link with cancer, remain a topic of controversy and require further investigation (13).

Therefore, the present umbrella review was conducted to systematically summarize and evaluate evidence from published systematic reviews and meta-analyses of observational studies to determine whether there is a significant association between artificial sweetener consumption and the risk of cancer. Importantly, the review includes all sources of artificial sweeteners—not limited to ASBs—to provide a comprehensive assessment of their potential link to cancer.

## Methods

### Study protocol

This study was carried out according to the guidelines of the Preferred Reporting Items for Systematic Reviews and Meta-Analyses (PRISMA) to ensure a systematic and rigorous approach (18). A comprehensive search was conducted in prominent international scientific databases, namely PubMed, Scopus, EMBASE, and Web of Science, to identify relevant articles. The search encompassed all articles available in each database from its inception up to January 2025. The search was restricted to English-language publications and focused on identifying meta-analyses that examined the relationship between artificial sweetener consumption and cancer risk. Key terms used included: (“*Sweetening Agents*” *OR* “*Artificial Sweeteners*” *OR* “*Non-Nutritive Sweeteners*” *OR* “*Stevia*” *OR* “*Aspartame*” *OR* “*Saccharin*” *OR* “*Cyclamates*” *OR* “*Sucralose*” *OR* “*Acesulfame*”*) AND (*“*Neoplasms*” *OR* “*Carcinoma*” *OR* “*Cancer*”*) AND (*“*Meta-analysis*”). A detailed and repeatabe search strategy for PubMed database is provided in Supplementary material S1.

### Inclusion and exclusion criteria

This umbrella meta-analysis included observational meta-analyses investigating the association of any type of artificial sweeteners and cancer risk providing risk ratio (RR), odds ratio (OR), or hazard ratio (HR) along with their corresponding confidence intervals (CI). Additionally, studies conducted *in vitro, in vivo*, and *ex vivo*, as well as case reports, quasi-experimental studies, controlled clinical trials were excluded. The term “dataset” in this study refers to each independent analysis reported within a meta-analysis, including overall estimates and subgroup analyses (e.g., by cancer type, study design, or exposure level). Thus, some individual meta-analyses contributed multiple datasets when separate pooled estimates were provided for different subgroups or outcomes.

### Methodological quality assessment and data extraction

Two independent reviewers assessed the methodological quality of the included articles using the Assessing the Methodological Quality of Systematic Reviews 2 (AMSTAR2) questionnaire (19). The AMSTAR 2 tool includes 16 items that are answered with “Yes,” “Partial Yes,” “No,” or “Not a Meta-analysis.” These items are divided into four categories: “Critically low quality,” “Low quality,” “Moderate quality,” and “High quality.” If any discrepancies arose, the first author was consulted to achieve a consensus. A score of 7 or higher indicated that a meta-analysis was of high quality. To ensure clarity, we would like to emphasize that the AMSTAR 2 includes critical items. If any of these critical items are answered with “No,” the meta-analysis cannot be considered of “High quality”, regardless of the overall score

Four reviewers independently also extracted the following data from the included meta-analyses: year of publication, sample size, study location, type of artificial sweeteners, effect sizes (ESs) including HR, RR, OR, and corresponding CIs, which were subsequently recorded in an Excel spreadsheet.

### Data synthesis and statistical analysis

Random-effects model with restricted maximum likelihood method (REML) (20) was employed to estimate the pooled ES and its corresponding 95% CI. To assess heterogeneity, the *I*^2^ statistic and Cochrane's Q-test were utilized. Heterogeneity was considered substantial if the *I*^2^ value exceeded 50% or if the *p*-value for the Q-test was < 0.1 (20). Subgroup analyses were conducted based on predefined variables, such as the types of cancer, effect size, study design and source of artificial sweetener. Sensitivity analysis was performed to evaluate the impact of individual study's removal on the overall effect size. Begg's and Egger's tests and visual inspection of funnel plot were performed to assess publication bias. In case of presence of publication bias, trim and fill analysis was carried out to simulate an effect size considering publication with inserting new hypothetical studies. All statistical analyses were conducted using Stata version 16 (Stata Corporation, College Station, TX, US), and a *p*-value < 0.05 was considered statistically significant.

## Results

### Summary of literature review

**Figure 1:**
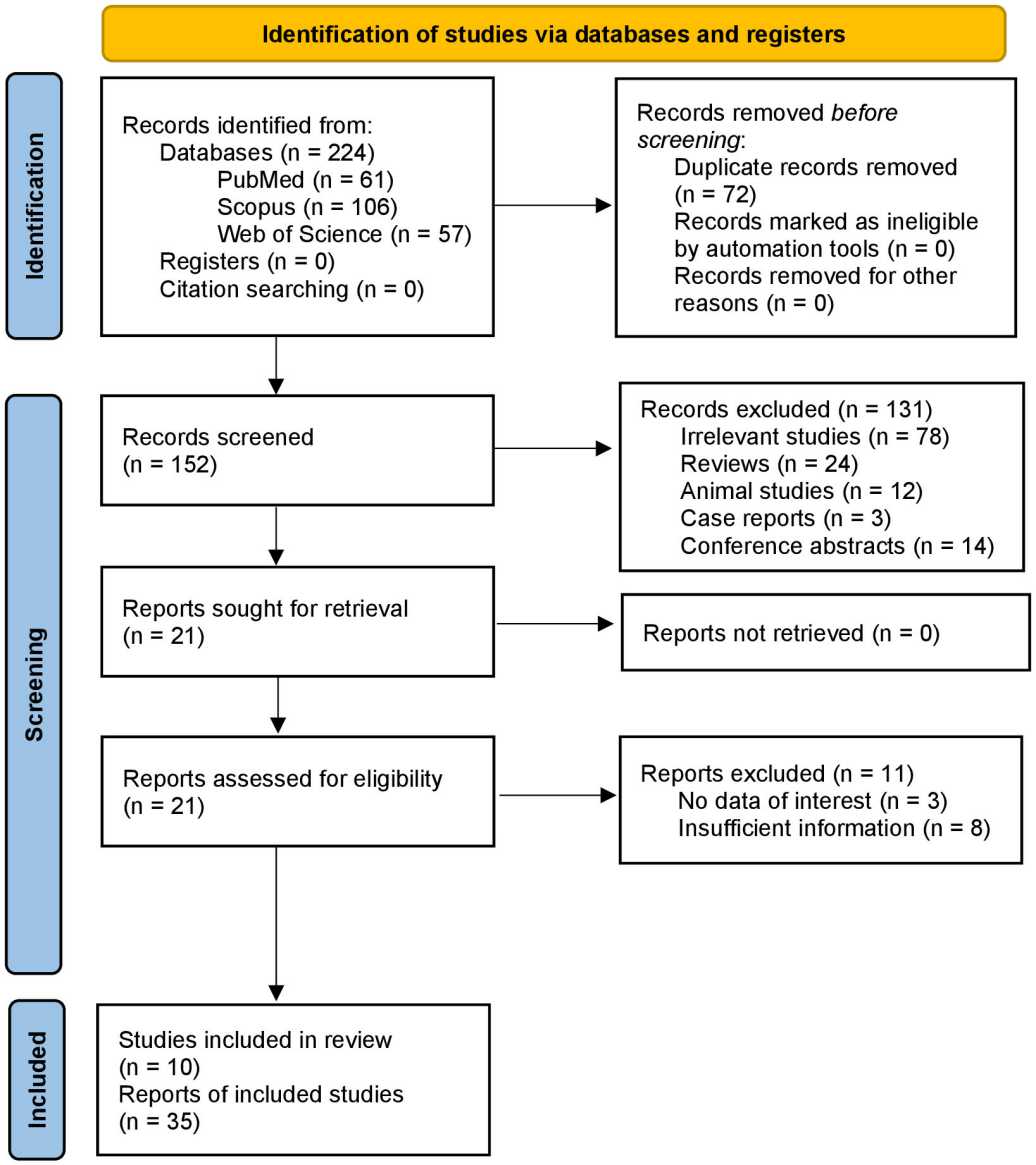
PRISMA flow diagram illustrating the study selection process. The diagram summarizes the number of records identified through database searches, duplicates removed, titles and abstracts screened, full-text articles assessed for eligibility, and meta-analyses included in the umbrella review. Reasons for full-text exclusions are also indicated.

First, we retrieved 224 articles by searching the databases. Second, 152 studies relevant to the intake of artificial sweeteners and cancer risk were remained after deduplication. Then, after evaluating the titles and abstracts, 131 articles were excluded. Additionally, 11 studies were excluded after full-text screening. The selection process and reasons for exclusion are presented in a flow diagram (Figure 1). Finally, a total of 10 studies with 35 datasets were regarded as eligible for the umbrella review. We have also provided the list of these excluded studies in the full-text evaluation stage and the reasons in the Supplementary material S2.

### Characteristics of the included meta-analyses

The characteristics of 10 meta-analyses with 35 datasets are presented in Table 1. Of these datasets, eight were from cohort studies, six from case-control studies, three included both study types, and 18 incorporated all types of observational studies. A total of four studies were conducted to examine the association between intake of artificial sweeteners and the potential risk of getting pancreatic cancer (16, 17, 21, 22). Two studies were conducted to investigate the risk of gastric cancer (16, 22). Additionally, two studies focused on colorectal cancer (16, 21), one study examined oesophageal cancer (16), another study explored breast cancer (21), and one study investigated prostate cancer (21). Furthermore, there were four supplementary studies conducted, specifically focusing on cancer in the digestive system (23), gynecological cancer (23), genitourinary cancer (23), and endometrial cancer (21), in addition to a study on hematopoietic cancer (21). In addition, a singular study focused exclusively on bladder cancer (24), whereas five datasets collectively explored multiple types of cancers (1, 16, 23).

**Table 1:** Study characteristics of the included studies.

**First author**	**Year**	**Study type**	**Source of artificial sweeteners**	**Outcome**	**Age**	**Type of analyzed effect size**	**Effect size (95% CI)**	**Follow-up**	**Number of included studies**
Xia Ye (a)	2023	All studies	Artificial sweeteners (overall)	Breast cancer	NR	OR	0.98 (0.94, 1.03)	16	11
Xia Ye (b)	2023	All studies	Artificial sweeteners (low dose)	Breast cancer	NR	OR	1.01 (0.95, 1.07)	17	2
Xia Ye (c)	2023	All studies	Artificial sweeteners (middle dose)	Breast cancer	NR	OR	0.98 (0.93, 1.02)	17	2
Xia Ye (d)	2023	All studies	Artificial sweeteners (high dose)	Breast cancer	NR	OR	0.88 (0.74, 1.06)	16	3
Huiping Li	2023	All studies	Non-nutritional	Endometrial cancer	NR	OR	0.90 (0.81, 1.01)	NR	11
Bei Pan (a)	2023	All studies	Artificial sweeteners	Overall cancer	48	RR	0.96 (0.86, 1.08)	9	2
Bei Pan (b)	2023	All studies	Artificial sweeteners	Breast cancer	45	RR	0.95 (0.8, 1.12)	14	3
Bei Pan (c)	2023	All studies	Artificial sweeteners	Colorectal cancer	49	RR	0.93 (0.78, 1.1)	8	2
Bei Pan (d)	2023	All studies	Artificial sweeteners	Multiple myeloma	51.5	RR	1.14 (0.81, 1.6)	22	2
Bei Pan (e)	2023	All studies	Artificial sweeteners	Non-Hodgkin lymphoma	60	RR	1.00 (0.9, 1.11)	18	3
Bei Pan (f)	2023	All studies	Artificial sweeteners	Pancreatic cancer	55	RR	1.03 (0.96, 1.1)	16	3
Bei Pan (g)	2023	All studies	Artificial sweeteners	Prostate cancer	60	RR	0.93 (0.69, 1.26)	10	2
Tongxin Yin (a)	2022	Prospective Studies	Artificially sweetened beverage	Pancreatic cancer	NR	RR	1.10 (0.92, 1.31)	11.5	4
Tongxin Yin (b)	2022	Prospective Studies	Artificially sweetened beverage	Colorectal cancer	NR	RR	0.78 (0.62, 0.99)	13.5	3
Tongxin Yin (c)	2022	Prospective Studies	Artificially sweetened beverage	Breast cancer	NR	RR	0.99 (0.9, 1.08)	12.5	4
Tongxin Yin (d)	2022	Prospective Studies	Artificially sweetened beverage	Prostate cancer	NR	RR	1.06(0.69, 1.62)	11	2
Tongxin Yin (e)	2022	Prospective Studies	Artificially sweetened beverage	Hematopoietic cancer	NR	RR	1.08 (0.88, 1.32)	13	4
Tongxin Yin (f)	2022	Prospective Studies	Artificially sweetened beverage	Endometrial cancer	NR	RR	0.81 (0.64, 1.03)	15	2
Alfred Jatho (a)	2021	All studies	Artificially sweetened soft drinks	Pancreatic cancer	60	OR/RR/HR	1.13 (0.85, 1.5)	NR	8
Alfred Jatho (b)	2021	All studies	Artificially sweetened soft drinks	Liver cancer	60	OR/RR/HR	1.28 (1.03, 1.58)	NR	3
Alfred Jatho (d)	2021	All studies	Artificially sweetened soft drinks	Colorectal cancer	60	OR/RR/HR	0.98 (0.79, 1.23)	NR	9
Alfred Jatho (f)	2021	All studies	Artificially sweetened soft drinks	Overall cancer	60	OR/RR/HR	1.02 (0.92, 1.14)	NR	32
Alfred Jatho (e)	2021	Case control Studies	Artificially sweetened soft drinks	Overall cancer	60	OR/RR/HR	0.95 (0.82, 1.11)	NR	21
Alfred Jatho (f)	2021	Cohort	Artificially sweetened soft drinks	Overall cancer	60	OR/RR/HR	1.14 (0.97, 1.33)	NR	17
Alfred Jatho (g)	2021	All studies	Artificially sweetened soft drinks	Esophageal cancer	60	OR/RR/HR	0.88 (0.69, 1.13)	NR	9
Alfred Jatho (h)	2021	All studies	Artificially sweetened soft drinks	Gastric cancer	60	OR/RR/HR	1.00 (0.79, 1.27)	NR	9
Adam Tepler (a)	2021	Cohort and case-control studies	Artificial sweeteners	Pancreatic cancer	NR	OR	1.04 (0.95, 1.13)	NR	9
Adam Tepler (b)	2021	Cohort and case-control studies	Artificial sweeteners	Luminal gastrointestinal cancer	NR	OR	0.81 (0.68, 0.96)	NR	7
Fjorida Llaha	2021	Cohort and case-control studies	Artificially sweetened beverage	Pancreatic cancer	55	RR	1.07 (0.77, 1.48)	NR	5
Liping Liu (a)	2021	Case–control studies	Artificial sweeteners	Overall cancer	60	OR	0.91 (0.75, 1.11)	NR	19
Liping Liu (b)	2021	Case–control studies	Artificial sweeteners	Genitourinary cancer	60	OR	1.06 (0.85, 1.31)	NR	11
Liping Liu (c)	2021	Case–control studies	Artificial sweeteners	Digestive system cancer	60	OR	0.73 (0.45, 1.17)	NR	5
Liping Liu (d)	2021	Case–control studies	Artificial sweeteners	Gynecological cancer	60	OR	0.7(0.42, 1.17)	NR	3
Shoumeng Yan	2022	Prospective Studies	Artificial sweeteners	Overall cancer	NR	HR	1.04 (0.99, 1.08)	16	26
Ingrid Toews	2019	Case–control studies	Non-sugar sweeteners	Bladder cancer	NR	OR	1.03 (0.84, 1.25)	NR	10

NR, Not reported; RR, risk ratio; OR, odds ratio; HR, hazard ratio.

### Association between intake of artificial sweeteners and various cancer risks

**Figure 2:**
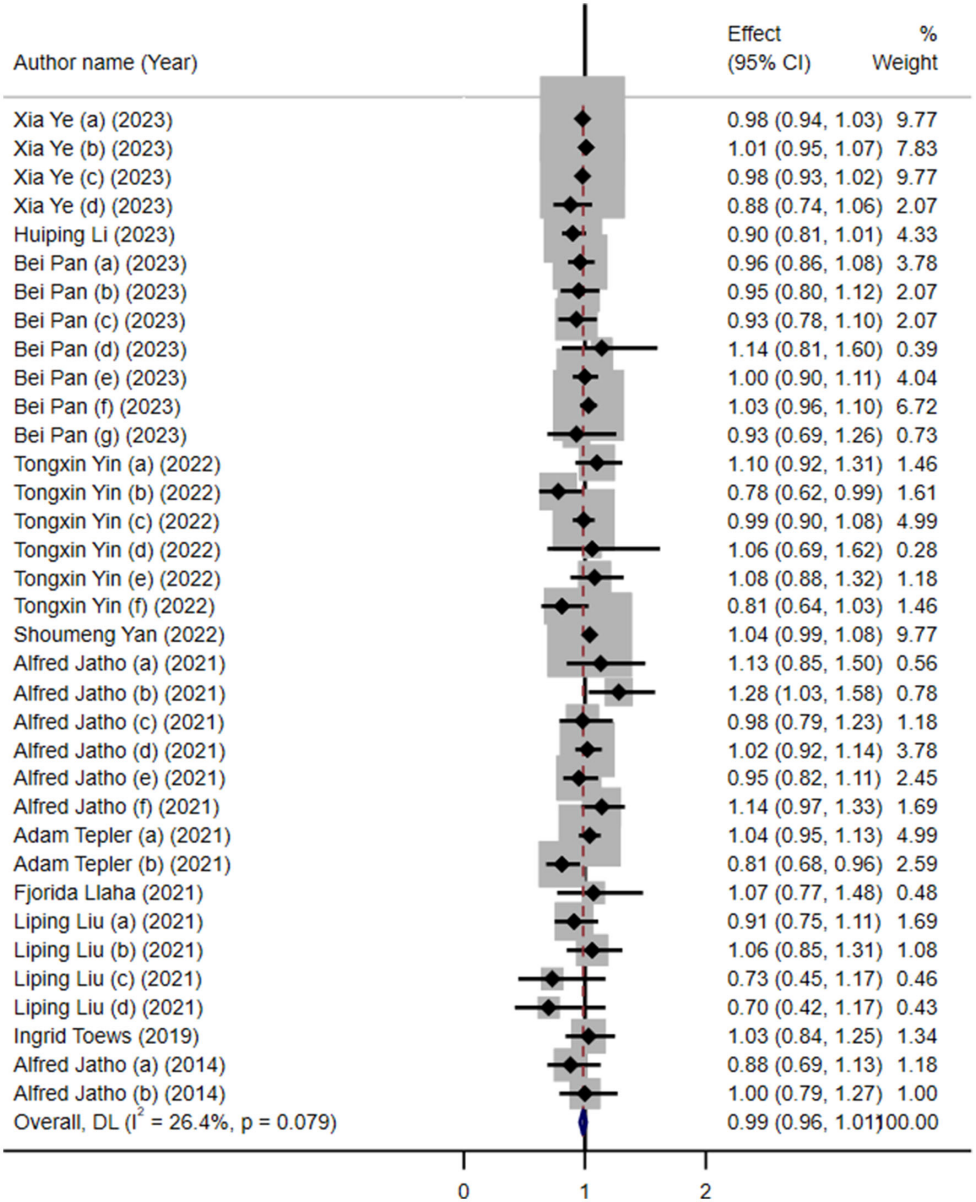
Forest plot depicting the overall association between artificial sweetener intake and cancer risk across 35 datasets included in the umbrella review. Effect estimates are shown as risk ratios (RRs) with 95% confidence intervals (CIs), calculated using a random-effects model. Each horizontal line represents a single dataset's RR and CI, and the diamond indicates the pooled estimate. Heterogeneity was assessed using the *I*^2^ statistic.

The results showed no significant association between artificial sweetener intake and overall cancer risk (RR: 0.99; 95% CI: 0.96–1.01; *I*^2^ = 26.4%; Figure 2). As summarized in Table 2, our subgroup analysis by cancer type revealed no significant associations, except for gynecological cancers, where a higher intake was linked to a reduced risk (RR: 0.87, 95% CI: 0.79, 0.96; *I*^2^ = 0%). The intake of ASBs (RR: 0.98; 95% CI: 0.96–1.01) and artificial sweeteners in general (RR: 1.00; 95% CI: 0.94–1.06) showed no significant association with cancer risk, both with low heterogeneity (Table 2).

**Table 2:** Subgroup analyses for the association of artificial sweeteners and cancer risk.

**Subgroup category**	**Effect size number**	**ES (95% CI)**	***I*^2^ (%)**	***p*-value (heterogeneity)**
**Association between intake of artificial sweeteners and cancer risk**				
Overall	35	0.99 (0.96, 1.01)	26.4	0.07
**Cancer type**				
Pancreatic cancer	5	1.04 (0.99, 1.09)	0.0	0.94
Breast cancer	6	0.98 (0.96, 1.01)	0.0	0.76
Gastrointestinal cancer	8	0.91 (0.81, 1.01)	0.07	0.45
All cancer	6	1.02 (0.97, 1.06)	15.6	0.31
Gynecological cancer	3	0.87 (0.79, 0.96)	0.0	0.47
Genitourinary cancer	4	1.02 (0.89, 1.15)	0.0	0.91
Hematopoietic cancer	3	1.02 (0.93, 1.11)	0.0	0.67
**Source of Sweeteners**				
Artificially sweetened beverage	20	0.98 (0.96, 1.01)	28.7	0.11
Artificial sweeteners	15	1 (0.94, 1.06)	28.2	0.14
**Study type**				
Prospective study	8	1.00 (0.92, 1.08)	52.6	0.03
Case-control study	6	0.94 (0.86, 1.03)	0.0	0.44
Cohort and case-control	3	0.96 (0.77, 1.14)	73.7	0.02
All studies	18	0.99 (0.96, 1.01)	0.0	0.60
**Effect size**				
RR	14	0.99 (0.95, 1.02)	3.1	0.41
OR/RR/HR	8	1.03 (0.95, 1.10)	14.4	0.31
OR	12	0.96 (0.93, 1)	36.8	0.09
HR	1	1.04 (0.99, 1.08)	NA	NA
**Study quality**				
Low quality	9	0.98 (0.94, 1.01)	31	0.16
High quality	17	0.99 (0.95, 1.02)	2.9	0.42
Moderate quality	9	1.04 (0.99, 1.09)	10	0.35

n, number of effect sizes; NA, not applicable; ES, Effect size; RR, risk ratio; OR, odds ratio; HR, hazard ratio; CI, confidence interval.

When stratified by study design (Table 2), we found no significant association between artificial sweetener intake and cancer risk across prospective studies (RR: 1.00; 95% CI: 0.92–1.08; *I*^2^ = 52.6%; *p* = 0.03), case-control studies (RR: 0.94; 95% CI: 0.86–1.03; *I*^2^ = 0.0%; *p* = 0.44), and cohort-case-control studies (RR: 0.96; 95% CI: 0.77–1.14; *I*^2^ = 73.7%; *p* = 0.02). The overall pooled estimate across all study types (RR: 0.99; 95% CI: 0.96–1.01; *I*^2^ = 0.0%; *p* = 0.60) indicated no significant association (Table 2).

Furthermore, a subgroup analysis based on the methodological quality of the included reviews, with detailed quality scores in Table 3, also revealed no statistically significant associations (Table 2). High-quality studies (*n* = 17) reported a pooled RR of 0.99 (95% CI: 0.95–1.02; *I*^2^ = 2.9%), low-quality studies (*n* = 9) showed a similar estimate (RR: 0.98; 95% CI: 0.94–1.01; *I*^2^ = 31%), and moderate-quality studies (*n* = 9) showed a slightly elevated, but still non-significant association (RR: 1.04; 95% CI: 0.99–1.09; *I*^2^ = 10%). The consistency across quality levels supports the robustness of the overall null association (Table 2).

**Table 3:** Results of assessment of the methodological quality of the included meta-analyses.

**First author, year**	**Q1**	**Q2**	**Q3**	**Q4**	**Q5**	**Q6**	**Q7**	**Q8**	**Q9**	**Q10**	**Q11**	**Q12**	**Q13**	**Q14**	**Q15**	**Q16**	**Overall**
Alfred Jatho 2021	Yes	Yes	Yes	Partial Yes	Yes	Yes	Yes	Yes	Yes	No	Yes	Yes	Yes	Yes	Yes	No	Moderate
Adam Tepler 2021	Yes	Yes	Yes	Partial Yes	Yes	Yes	Yes	Yes	Yes	No	Yes	Yes	No	Yes	Yes	Yes	Low
Fjorida Llaha 2021	Yes	Yes	Yes	Partial Yes	Yes	Yes	Yes	Yes	Yes	No	Yes	Yes	Yes	Yes	No	Yes	Low
Tongxin Yin 2022	Yes	Yes	Yes	Partial Yes	Yes	Yes	Yes	Yes	Yes	No	Yes	Yes	Yes	Yes	Yes	Yes	High
Liping Liu 2021	Yes	Yes	Yes	Partial Yes	Yes	Yes	Yes	Yes	Yes	No	Yes	Yes	Yes	Yes	Yes	Yes	High
Shoumeng Yan 2022	Yes	Yes	Yes	Partial Yes	Yes	No	Yes	Yes	Yes	No	Yes	Yes	Yes	Yes	Yes	Yes	Moderate
Ingrid Toews 2019	Yes	Yes	Yes	Partial Yes	Yes	Yes	Yes	Yes	Yes	No	Yes	Yes	Yes	Yes	No	Yes	Low
Xia Ye 2023	Yes	Yes	Yes	Partial Yes	Yes	Yes	Yes	Yes	No	Yes	Yes	No	No	Yes	Yes	Yes	Low
Huiping Li 2023	Yes	Yes	Yes	Partial Yes	Yes	Yes	Yes	Yes	No	Yes	Yes	No	No	Yes	Yes	Yes	Low
Bei Pan 2023	Yes	Yes	Yes	Partial Yes	Yes	Yes	Yes	Yes	Yes	Yes	Yes	Yes	Yes	Yes	Yes	Yes	High

Q1- Did the research questions and inclusion criteria for the review include the components of PICO? Q2- Did the report of the review contain an explicit statement that the review methods were established prior to the conduct of the review, and did the report justify any significant deviations from the protocol? Q3- Did the review authors explain their selection of the study designs for inclusion in the review? Q4- Did the review authors use a comprehensive literature search strategy? Q5- Did the review authors perform study selection in duplicate? Q6- Did the review authors perform data extraction in duplicate? Q7- Did the review authors provide a list of excluded studies and justify the exclusions? Q8- Did the review authors describe the included studies in adequate detail? Q9- Did the review authors use a satisfactory technique for assessing the risk of bias (RoB) in individual studies that were included in the review? Q10- Did the review authors report on the sources of funding for the studies included in the review? Q11- If meta-analysis was performed, did the review authors use appropriate methods for the statistical combination of results? Q12- If a meta-analysis was performed, did the review authors assess the potential impact of RoB in individual studies on the results of the meta-analysis or other evidence synthesis? Q13- Did the review authors account for RoB in individual studies when interpreting/discussing the review results? Q14- Did the review authors provide a satisfactory explanation for and discussion of any heterogeneity observed in the review results? Q15- If they performed quantitative synthesis, did the review authors conduct an adequate investigation of publication bias (small-study bias) and discuss its likely impact on the review results? Q16- Did the review authors report any potential sources of conflict of interest, including any funding they received for conducting the review?

Analysis by statistical measure showed consistent results. Studies using RR reported a pooled estimate of 0.99 (95% CI: 0.95–1.02; *I*^2^ = 3.1%; *p* = 0.41). Those combining OR, RR, or HR showed an estimate of 1.03 (95% CI: 0.95–1.10; *I*^2^ = 14.4%; *p* = 0.31). For the OR alone, the estimate was 0.96 (95% CI: 0.93–1.00; *I*^2^ = 36.8%; *p* = 0.09), and for the HR alone, it was 1.04 (95% CI: 0.99–1.08; *I*^2^ = 0.0%; *p* = 0.001). None indicated a significant association (Table 2).

**Figure 3:**
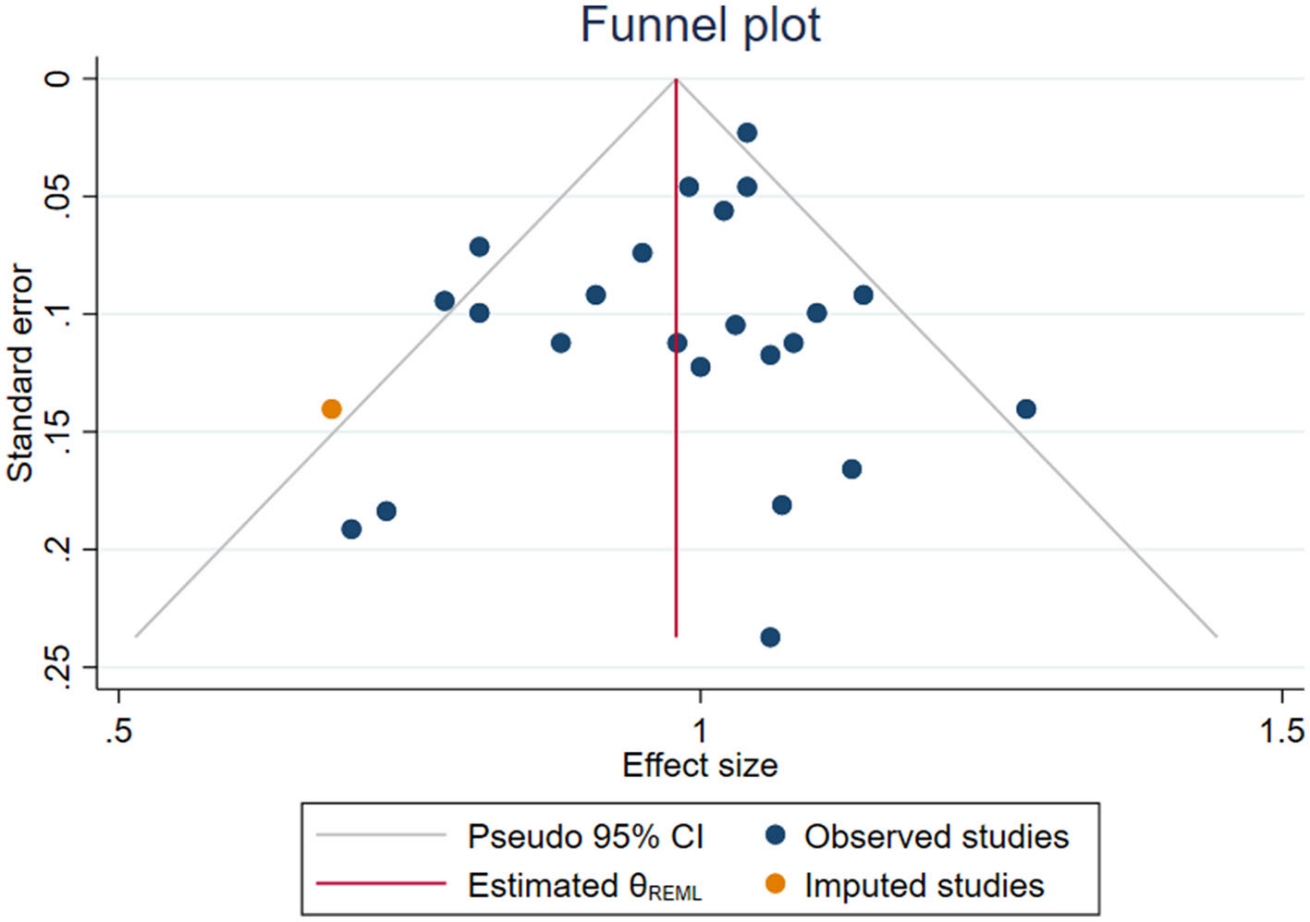
Funnel plot assessing potential publication bias for the association between artificial sweetener intake and cancer risk. Each dot represents an individual dataset included in the umbrella review. The plot displays effect sizes against their standard errors. Symmetry was evaluated using Egger's and Begg's statistical tests, both of which indicated no significant publication bias.

Furthermore, the findings of the sensitivity analysis remained consistent with the overall result, suggesting that the overall association between artificial sweetener intake and cancer risk is robust to variations in the data (Supplementary material S3). No significant small-study effects were observed based on Egger's and Begg's tests (*p* = 0.30 and *p* = 0.87, respectively). Furthermore, no evidence of publication bias was detected through the visual inspection of the funnel plot (Figure 3).

### Methodological quality

The methodological quality of the eligible meta-analyses was assessed by a validated AMSTAR 2 tool. The overall and detailed AMSTAR 2 scores for each meta-analysis are provided in Table 3. Among the 10 included studies, three were of high quality (21, 23, 25), two were of moderate quality (1, 16), and five were of low quality (17, 22, 24, 26, 27).

## Discussion

This umbrella review synthesizes evidence from 10 meta-analyses encompassing 35 datasets to evaluate the association between artificial sweetener intake and the risk of developing various cancer types. The comprehensive nature of this study provides a broad perspective on the current evidence base, offering an integrative assessment of both site-specific and overall cancer risks. The synthesis of available data showed no overall significant association between artificial sweetener consumption and the risk of total cancer or most site-specific cancers, such as colorectal, pancreatic, and gastric cancers. These findings were generally consistent across study designs, exposure types, and statistical approaches, reinforcing the overall neutrality of the observed association. However, a notable exception was observed in the subgroup of gynecological cancers, where a inverse association was identified. This inverse association is noteworthy, although it should be interpreted cautiously. One possible explanation may involve hormonal or metabolic pathways influenced by artificial sweeteners. For example, some low-calorie sweeteners have been shown to alter insulin sensitivity and estrogen signaling (8), which could theoretically influence gynecologic cancer development. Additionally, gut microbiota changes induced by artificial sweeteners may affect systemic inflammation or hormonal regulation, which are relevant to gynecological cancer risk. However, due to limited mechanistic evidence in humans (28), these hypotheses remain speculative. From a methodological standpoint, this result was based on only three datasets, with limited sample size compared to more extensively studied cancers like breast or colorectal cancer. As such, the apparent protective association may be influenced by chance, selective reporting, or residual confounding. Further site-specific, high-quality studies are needed to validate this finding and explore its underlying mechanisms. In contrast, mechanisms that might explain potential risks for other cancers, such as liver cancer as noted in some studies, have also been proposed.

The metabolism of specific sweeteners is a primary concern. Aspartame, for instance, is metabolized into methanol and subsequently into formaldehyde, a well-established Group 1 carcinogen known to be genotoxic and capable of damaging DNA. Chronic exposure to such a metabolite could, in theory, increase susceptibility to cancer in certain tissues like the liver (29).

Moreover, as with potential protective effects, the gut microbiota is also implicated in pathways that may increase risk. Sweetener-induced gut dysbiosis has been linked to increased intestinal inflammation and insulin resistance, both of which are recognized risk factors in the development of certain cancers, including hepatocellular carcinoma (30). This suggests a potential site-specific effect that warrants further investigation. The underlying mechanisms remain unclear, but this finding raises the possibility that certain biological pathways related to hormonal or reproductive systems might interact differently with artificial sweeteners. Such observations emphasize the importance of not assuming uniform effects of dietary components across all cancers.

The findings of our umbrella review are consistent with previous meta-analyses that explored site-specific associations, especially regarding breast cancer. A recent meta-analysis of observational studies similarly found no significant link between artificial sweetener intake and breast cancer risk, regardless of exposure levels. This alignment supports the robustness of the observed neutral association. However, unlike breast cancer, our review identified a significant inverse relationship with gynecological cancers, suggesting potential site-specific effects that warrant further research (26).

Beyond the cancers examined in our review, some individual studies have reported site-specific risks that fall outside the general pattern of neutrality. Notably, one study reported a 28% increase in liver cancer risk (16). While the precise mechanisms underlying the impact of artificial sweeteners on the liver remain unclear, several studies have provided evidence suggesting that the consumption of artificial sweeteners can lead to alterations in the intestinal microbiota, insulin resistance, oxidative stress, and liver inflammation, factors that could contribute to the development of liver cancer. (8, 28, 31–33). Soffritti et al. (34) reported that aspartame led to the development of cancerous tumors in the liver and lungs of mice. Methanol, a component of aspartame, is metabolized into formaldehyde, a known carcinogen (35–37). Formaldehyde has genotoxic effects and can damage DNA by forming formaldehyde adducts, which increase the risk of chromosomal mutations due to DNA-protein cross-linking. (38). Therefore, chronic intake of artificially sweetened soft drinks may increase susceptibility to hepatocellular carcinoma in humans. One possible explanation for the significance of this finding may be the longer follow-up period in liver cancer cohorts, which allows for better detection of associations over time. Another possibility could be the limited number of studies and small sample sizes, which warrant further investigation.

To further examine the influence of study quality on pooled estimates, we conducted a subgroup analysis stratified by methodological quality. Notably, high-quality studies—those most rigorously conducted—produced results consistent with the overall null association, and exhibited very low heterogeneity. The pooled RR from these studies was 0.99 (95% CI: 0.95–1.02; *I*^2^ = 2.9%), nearly identical to the main result. Similarly, low-quality studies showed no significant association, while moderate-quality studies showed a slightly elevated risk (RR: 1.04), albeit still statistically non-significant. These findings suggest that the inclusion of low-quality studies did not bias the overall outcome. However, it is crucial to emphasize that while the inclusion of lower-quality studies did not appear to bias the point estimate in this analysis, their prevalence (five of 10 studies) inherently weakens the overall strength of the evidence. A conclusion built upon a foundation where half the evidence is methodologically weak must be interpreted with significant caution. This fragility underscores the urgent need for more methodologically rigorous primary studies and meta-analyses in this field.

It is also important to address the significant heterogeneity observed in some subgroup analyses, despite the low overall heterogeneity. For instance, the analysis of combined cohort and case-control studies exhibited substantial heterogeneity (*I*^2^ = 73.7%). This variability may stem from several sources. Methodologically, combining different study designs, each with its own inherent biases and approaches to data collection, can introduce statistical inconsistency. Furthermore, the limited number of datasets in this subgroup (three) means that differences in population characteristics, exposure assessment methods, or the extent of adjustment for key confounding variables (such as smoking, physical activity, or overall dietary patterns) could have a magnified impact on the pooled estimate. The primary studies within this subgroup may have also focused on different cancer types or sweetener exposures, further contributing to the observed heterogeneity. This highlights that while the overall findings are robust, caution is warranted when interpreting subgroups with high statistical variance, reinforcing the need for more standardized research in the future.

Additionally, low methodological quality in many of the included studies may have introduced confounding and biased the results. For instance, many studies failed to adjust for key dietary factors such as fruit intake, which could confound the observed associations. Despite subgroup analyses based on study design showing no significant association, the evidence still highlights the need for more high-quality prospective cohort studies to validate the observed associations.

According to the World Health Organization (WHO) (39), there is a potential link between aspartame consumption and increased cancer risk, though it remains safe at doses below 40 mg/kg of body weight. While aspartame has been classified as Group 2B by the International Agency for Research on Cancer (IARC), indicating that it is possibly carcinogenic to humans based on limited evidence (39), it simultaneously emphasized that it remains safe at intake levels below 40 mg/kg body weight. It is important to note that the WHO evaluation was based on hazard identification, focusing primarily on experimental animal data and mechanistic evidence, whereas our umbrella review synthesized observational epidemiologic data on artificial sweeteners more broadly, without distinguishing between specific compounds. Additionally, most of the included studies did not quantify intake levels precisely, nor stratify by sweetener subtype. Therefore, the scope, exposure definitions, and methodological frameworks of our review differ from those of the WHO's risk assessment. This distinction should be considered when interpreting our findings and comparing them with regulatory assessments. Animal studies, including the work by Landrigan and Straif (40) have demonstrated that consuming high doses of aspartame (e.g., 100 mg/kg) significantly increases cancer risk levels far above typical human exposure.

While a consistent link between artificial sweeteners and cancer has been observed in animal studies, most human studies, including the present investigation, have not found statistically significant associations (34, 41–44). This discrepancy could be due to physiological differences between humans and animals, including differences in gastrointestinal structure and function that affect bioavailability.

The results of the sensitivity analysis confirmed the stability and reliability of the main findings, indicating that the observed association between artificial sweetener intake and cancer risk remained consistent even when individual studies were systematically excluded. This consistency highlights the robustness of the pooled estimates across various scenarios. Additionally, the absence of small-study effects based on Egger's and Begg's tests further supports the credibility of the results. Visual inspection of the funnel plot also revealed no signs of publication bias, strengthening the confidence in the overall conclusion of the meta-analysis.

A key point of this umbrella review is that a formal dose-response analysis was not conducted, as it falls beyond the methodological scope of synthesizing aggregate data from existing meta-analyses. A qualitative synthesis is also challenging because dose-response trends were not uniformly assessed in the included reviews. However, examining the available dose-response data provides important nuances to our main finding of a null overall association. Notably, two separate meta-analyses reported a consistent, positive linear dose-response relationship between ASB consumption and the risk of leukemia, with one study finding a 15% increased risk per daily serving (21) and another a 16% increased risk per 250 ml/day (25). This specific, dose-dependent risk contrasts with the findings for overall cancer and breast cancer, where dose-response analyses found no significant associations at any intake level. Furthermore, the relationship is not always linear, as one analysis suggested a potential protective effect for low-dose, but not high-dose, non-nutritional sweetener intake on endometrial cancer risk (27). These varied findings highlight that while the aggregate evidence does not support a link with overall cancer, specific dose-dependent risks (leukemia) or non-linear effects may exist for certain cancers, underscoring the need for more targeted research.

A key strength of this umbrella review is its comprehensive synthesis of existing meta-analyses on artificial sweetener intake and cancer risk. Unlike prior individual meta-analyses that focused on specific cancer types or sweetener sources, this review provides a broader and more integrated perspective by evaluating the consistency and quality of evidence across multiple datasets. Through subgroup analyses, sensitivity tests, and AMSTAR 2-based methodological appraisal, we identified patterns of association, sources of heterogeneity, and areas where the evidence is limited or uncertain. This approach enhances the clinical interpretability of existing findings and underscores the need for future high-quality, standardized studies. Our results provide a useful framework for researchers to design more robust meta-analyses and for clinicians and policymakers to interpret the evidence base more cautiously and holistically. This study has several limitations that warrant consideration. The included meta-analyses varied in how they reported exposure assessment, reflecting differences in the original observational studies they synthesized. Most primary studies relied on self-reported dietary intake data, often obtained through food frequency questionnaires or dietary recalls, to assess the type, quantity, and frequency of artificial sweetener consumption. These methods are subject to recall bias and potential misclassification, and exposure definitions were not standardized across studies. This variability may have introduced heterogeneity and reduced the precision of pooled estimates, potentially diluting true associations or generating spurious findings. It is important to note that most of the meta-analyses included in this umbrella review reported that the original observational studies had adjusted for key confounding variables such as age, sex, and BMI. However, the extent and detail of adjustment for other important factors—such as smoking status, physical activity, and baseline health conditions—varied across studies, which may have contributed to heterogeneity and influenced the observed associations. Due to the aggregate nature of the data, we were unable to directly evaluate or stratify by more granular factors such as genetic background, comorbidities, or other lifestyle characteristics. Future meta-analyses utilizing individual participant data could enable more precise adjustment and exploration of potential effect modifiers. As evaluated using a standardized appraisal tool, some reviews demonstrated high methodological rigor, while others were of moderate to low quality. This inconsistency in review quality highlights the importance of cautious interpretation, especially when drawing conclusions from lower-quality evidence.

Another important limitation of this umbrella review is the inability to perform subgroup analyses by specific artificial sweetener types (e.g., aspartame, sucralose, saccharin). Although these compounds differ in their chemical structure, absorption, metabolism, and biological activity, none of the included meta-analyses provided disaggregated results based on individual sweeteners. As a result, we were unable to explore potentially distinct effects across sweetener types. This limitation highlights the need for future research to report results stratified by sweetener subtype, which may help clarify differential health effects and mechanistic pathways.

In light of these findings and limitations, we emphasize the need for further high-quality, prospective studies that incorporate accurate dietary assessments, dose-response analyses, and mechanistic investigations to clarify the causal pathways linking artificial sweetener intake to cancer risk. Another limitation is the potential for multiple comparison bias arising from the number of subgroup analyses conducted. As these analyses were exploratory and intended to examine consistency across strata rather than to test predefined hypotheses, formal statistical adjustments (e.g., Bonferroni correction) were not applied—consistent with common practice in meta-analyses. Therefore, findings from subgroup analyses, particularly those with marginal significance, should be interpreted with appropriate caution. Despite the noted limitations, we believe this umbrella review contributes to a more comprehensive understanding of the current evidence concerning artificial sweetener intake and its potential association with cancer risk. By integrating data across multiple cancer types and sweetener compounds, this study offers a nuanced perspective that can inform future research priorities and guide public health recommendations. Another limitation is the incomplete coverage of all cancer types in the included meta-analyses. While major sites such as breast, colorectal, and pancreatic cancers were examined, others were underrepresented. This may lead to an underestimation of overall cancer risk and increase the chance of false-negative results. Broader inclusion of cancer types in future meta-analyses is warranted.

## Conclusion

In conclusion, this umbrella review did not find evidence of a significant overall link between artificial sweetener intake and cancer risk. This general finding should be interpreted with caution, as it does not preclude potential risks or benefits associated with specific, individual sweetener compounds. However, possible associations with specific cancers, like gynecological malignancies, warrant further research. Given current limitations, more high-quality studies are needed to clarify these relationships. Moreover, our findings should be interpreted within the context of existing regulatory evaluations, such as the WHO's conclusion on aspartame, which are based on different methodological frameworks and exposure assessments.
