# Supplementary material for: The association of artificial sweeteners intake and risk of cancer: an umbrella meta-analysis
Source: Front Med (Lausanne). 2025 Sep 8;12:1647178. doi: 10.3389/fmed.2025.1647178 (PMC12450865; doi:10.3389/fmed.2025.1647178)
Supplement: Supplementary file 1 [file Supplementary_file_1.docx]

**Supplementary S1.** Search Strategy.

(((("Sweetening Agents"[Mesh] OR "Artificially Sweetened Beverages"[Mesh] OR "Non-Nutritive Sweeteners"[Mesh] OR "Stevia"[Mesh] OR "Saccharin"[Mesh] OR "Cyclamates"[Mesh]) OR "Aspartame"[Mesh]) OR ((((((((((("sweetening agent"[Title/Abstract]) OR ("artificial sweetener"[Title/Abstract])) OR ("non-nutritive sweetener"[Title/Abstract])) OR (sweetener[Title/Abstract])) OR (stevia[Title/Abstract])) OR (aspartame[Title/Abstract])) OR (saccharin[Title/Abstract])) OR (cyclamates[Title/Abstract])) OR (sucralose[Title/Abstract])) OR (acesulfame[Title/Abstract])) OR (sweetened[Title/Abstract]))) AND ((("Neoplasms"[Mesh]) OR "Carcinoma"[Mesh]) OR (((neoplasm*[Title/Abstract]) OR (carcinoma[Title/Abstract])) OR (cancer[Title/Abstract])))) AND (((meta-analysis[Publication Type]) OR (meta-analysis[Title/Abstract])) OR (meta[Title/Abstract])).
