# Supplementary material for: The association of artificial sweeteners intake and risk of cancer: an umbrella meta-analysis
Source: Front Med (Lausanne). 2025 Sep 8;12:1647178. doi: 10.3389/fmed.2025.1647178 (PMC12450865; doi:10.3389/fmed.2025.1647178)
Supplement: Supplementary file 2 [file Supplementary_file_2.docx]

**Supplementary S2**. The list of excluded articles during

| **Excluded articles** | **Reasons** |
| --- | --- |
| 1. Sweetened beverage consumption and risk of liver cancer by diabetes status: A pooled analysis | No data of interest |
| 1. Sweetened Beverages Consumption and Pancreatic Cancer: A Meta-Analysis | Insufficient information |
| 1. Modifiable risk factors for the prevention of bladder cancer: a systematic review of meta-analyses | Insufficient information |
| 1. Sweetened carbonated beverage consumption and cancer risk: meta-analysis and review | Insufficient information |
| 1. risk of colon cancer and coffee, tea, and Sugar-Sweetened Soft Drink intake: Pooled Analysis of Prospective cohort Studies | Insufficient information |
| 1. Soft drinks, sweetened beverages and risk of pancreatic cancer | Insufficient information |
| 1. Consumption of sugar-sweetened beverages and fruit juice and human cancer: a systematic review and dose-response meta-analysis of observational studies | Insufficient information |
| 1. Aspartame and Risk of Cancer: A Meta-analytic Review | No data of interest |
| 1. Association of Consumption of Sugar-Sweetened Beverages or Artificially Sweetened Beverages with Mortality: A Systematic Review and Dose–Response Meta-Analysis of Prospective Cohort Studies | Insufficient information |
| 1. The Dose-Response Associations of Sugar-Sweetened Beverage Intake with the Risk of Stroke, Depression, Cancer, and Cause-Specific Mortality: A Systematic Review and Meta-Analysis of Prospective Studies | No data of interest |
| 1. Association of soft drink and 100% fruit juice consumption with all-cause mortality, cardiovascular diseases mortality, and cancer mortality: A systematic review and dose-response meta-analysis of prospective cohort studies | Insufficient information |
