# Supplementary material for: The association of artificial sweeteners intake and risk of cancer: an umbrella meta-analysis
Source: Front Med (Lausanne). 2025 Sep 8;12:1647178. doi: 10.3389/fmed.2025.1647178 (PMC12450865; doi:10.3389/fmed.2025.1647178)

**Supplementary S3**. Leave-one-out sensitivity analysis of the overall association between artificial sweetener intake and cancer risk.


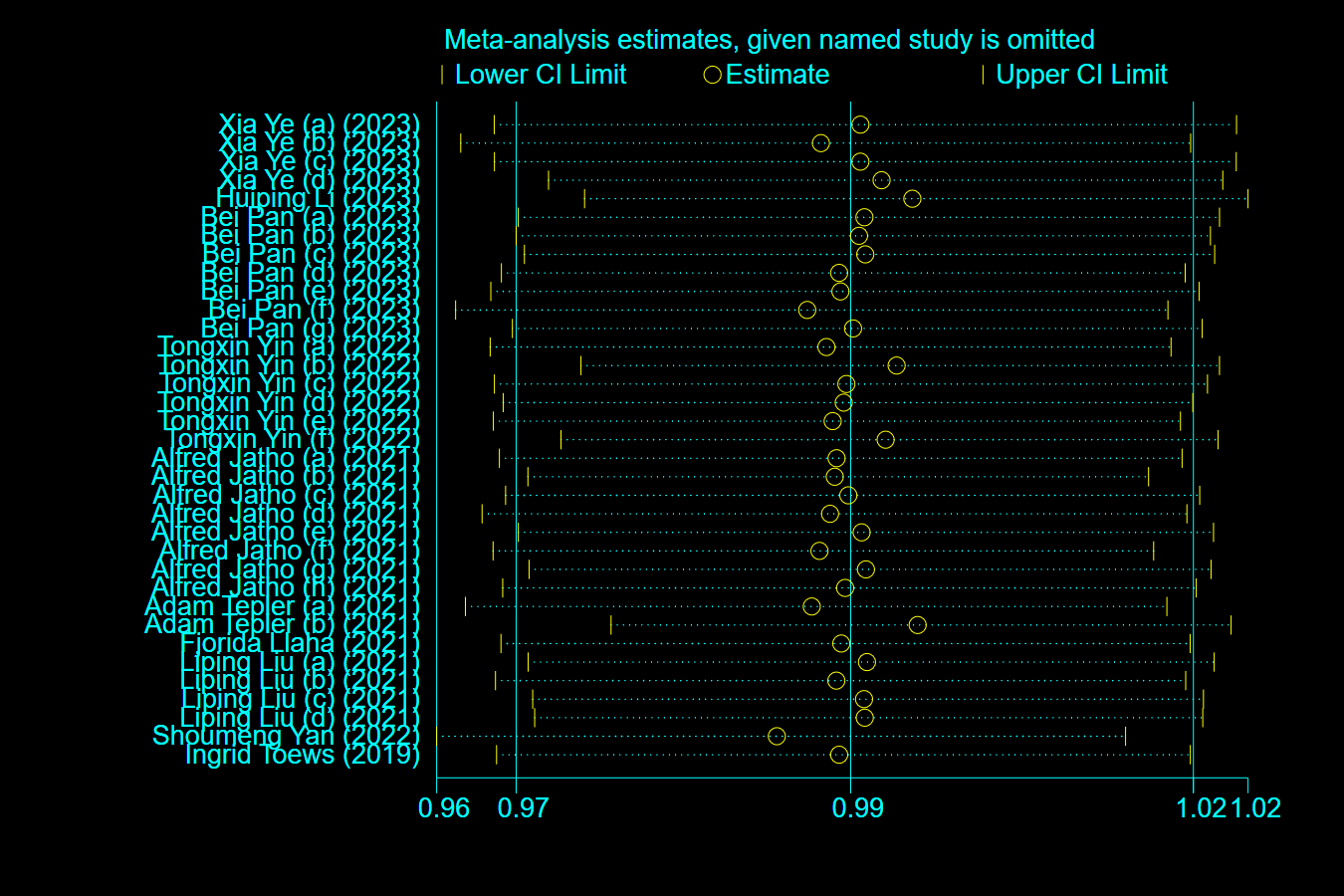

Supplement: Supplementary file 3 [file Supplementary_file_3.docx]
